# Goal-directed coagulation management of major trauma patients using thromboelastometry (ROTEM^®^)-guided administration of fibrinogen concentrate and prothrombin complex concentrate

**DOI:** 10.1186/cc8948

**Published:** 2010-04-07

**Authors:** Herbert Schöchl, Ulrike Nienaber, Georg Hofer, Wolfgang Voelckel, Csilla Jambor, Gisela Scharbert, Sibylle Kozek-Langenecker, Cristina Solomon

**Affiliations:** 1 Department of Anaesthesiology and Intensive Care, AUVA Trauma Hospital, Dr Franz-Rehrl-Platz 5, 5010 Salzburg, Austria; 2 Ludwig Boltzmann Institute for Experimental and Clinical Traumatology, Donaueschingenstrasse 13, A-1200 Vienna, Austria; 3 Institute for Research in Operative Medicine, University of Witten/Herdecke, Cologne-Merheim Medical Center, Ostmerheimer Strasse 200, 51109 Cologne, Germany; 4 Department of Anaesthesiology and Intensive Care, Munich University Hospital, Bavariaring 19, 80336 Munich, Germany; 5 Clinical Division B, Department of Anaesthesiology and General Intensive Care, Vienna Medical University, Spitalgasse 23, 1090 Vienna, Austria; 6 Department of Anaesthesiology and Intensive Care, Salzburger Landeskliniken SALK, 48 Müllner Hauptstrasse, 5020 Salzburg, Austria

## Abstract

**Introduction:**

The appropriate strategy for trauma-induced coagulopathy management is under debate. We report the treatment of major trauma using mainly coagulation factor concentrates.

**Methods:**

This retrospective analysis included trauma patients who received ≥ 5 units of red blood cell concentrate within 24 hours. Coagulation management was guided by thromboelastometry (ROTEM^®^). Fibrinogen concentrate was given as first-line haemostatic therapy when maximum clot firmness (MCF) measured by FibTEM (fibrin-based test) was <10 mm. Prothrombin complex concentrate (PCC) was given in case of recent coumarin intake or clotting time measured by extrinsic activation test (EXTEM) >1.5 times normal. Lack of improvement in EXTEM MCF after fibrinogen concentrate administration was an indication for platelet concentrate. The observed mortality was compared with the mortality predicted by the trauma injury severity score (TRISS) and by the revised injury severity classification (RISC) score.

**Results:**

Of 131 patients included, 128 received fibrinogen concentrate as first-line therapy, 98 additionally received PCC, while 3 patients with recent coumarin intake received only PCC. Twelve patients received FFP and 29 received platelet concentrate. The observed mortality was 24.4%, lower than the TRISS mortality of 33.7% (*P *= 0.032) and the RISC mortality of 28.7% (*P *> 0.05). After excluding 17 patients with traumatic brain injury, the difference in mortality was 14% observed versus 27.8% predicted by TRISS (*P *= 0.0018) and 24.3% predicted by RISC (*P *= 0.014).

**Conclusions:**

ROTEM^®^-guided haemostatic therapy, with fibrinogen concentrate as first-line haemostatic therapy and additional PCC, was goal-directed and fast. A favourable survival rate was observed. Prospective, randomized trials to investigate this therapeutic alternative further appear warranted.

## Introduction

Coagulopathy has been shown to be present in approximately 25 to 35% of all trauma patients on admission to the emergency room (ER) [1,2]. This represents a serious problem for major trauma patients and accounts for 40% of all trauma-related deaths [3]. Coagulopathy forces a strategy of early and rapid haemostatic treatment to prevent exsanguination. Fresh frozen plasma (FFP) is part of the massive transfusion protocols in most trauma centres [3-5], although its efficacy is uncertain. Massive transfusion protocols that favour a red blood cell (RBC):FFP ratio of 1:1 have shown conflicting results [6-14]. In addition, there are well-recognised risks associated with FFP administration in the trauma setting, such as acute lung injury, volume overload, and nosocomial infection [12,15-17]. According to the Serious Hazards of Transfusion (SHOT) report, the risk of transfusion-related acute lung injury (TRALI) following FFP transfusion is approximately 1:5000. The accumulation of 162 reports of TRALI to SHOT over eight years, and its implication in 36 deaths and 93 cases of major morbidity, has led to the recognition that TRALI is the most important cause of transfusion-associated mortality and morbidity [18].

It has been shown that the amount of fibrinogen administered to trauma patients correlates with survival [19]. Fibrinogen concentrate [20,21] and prothrombin complex concentrate (PCC) [22,23] have each previously been administered to trauma and surgical patients with success, albeit not in studies conducted exclusively in the trauma setting. However, there have not been any studies on the combined use of fibrinogen concentrate and PCC for prompt haemostatic therapy in trauma patients. The administration of coagulation factor concentrates may facilitate early and aggressive correction of coagulopathy by eliminating the time delay associated with cross-matching and thawing of FFP. Goal-directed haemostatic therapy with coagulation factor concentrates may also reduce transfusion of allogeneic blood products, which is desirable given their negative impact on the patient outcomes.

In recent years, viscoelastic methods that assess the speed of clotting and quality of the clot, such as thromboelastometry (ROTEM^®^, Tem International GmbH, Munich, Germany), have been successfully used to guide haemostatic therapy. Their application in the perioperative setting has been shown to decrease transfusion of allogeneic blood products and the costs associated with haemostatic management [24-26].

We investigated administration of fibrinogen concentrate as first-line haemostatic therapy in trauma patients with severe bleeding; additional PCC therapy was administered as required. These treatments were guided by thromboelastometry. Our hypothesis was that prompt, goal-directed coagulation treatment with coagulation factor concentrates may prove beneficial for patient outcomes. Observed mortality was compared with the mortality predicted by the trauma injury severity score (TRISS) and by the revised injury severity classification (RISC) score.

## Materials and methods

We studied patients who received five units or more of RBC within the first 24 hours after arrival at our trauma centre. Since 2001, ROTEM analysis has been part of our coagulation monitoring protocol for all trauma cases requiring the full trauma team in the ER. We use the ROTEM results to guide coagulation therapy, which mainly comprises coagulation factor concentrates. Approval from the local ethics committee was obtained for the retrospective collection of the data. As the coagulation analyses and the haemostatic therapy were part of the clinic's standard, the ethics committee waived the necessity to obtained informed written consent from the patients included in the analyses.

**Figure 1:**
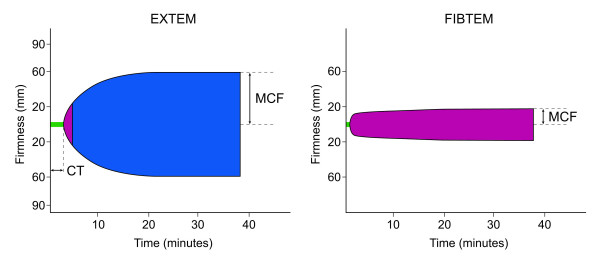
**The ROTEM^® ^analyses: EXTEM^® ^test (extrinsically activated test) and FibTEM^® ^test (fibrin clot obtained by platelet inhibition with cytochalasin D)**. The clotting time (CT (seconds)) represents the time from the start of the test until a clot firmness of 2 mm is detected; maximum clot firmness (MCF (mm)) represents the total amplitude of the clot.

The coagulation management was guided by thromboelastometry performed on the ROTEM device (Tem International GmbH, Munich, Germany). The method measures the viscoelastic properties of the clot and provides information on the speed of coagulation initiation, kinetics of clot growth, clot strength and breakdown [27]. The analyses are performed by pipetting 300 μl citrated whole blood and 20 μl 0.2 M calcium chloride with specific activators into a plastic cup. Measurement of coagulation in ROTEM is performed after the vertical immersion of a plastic pin into the blood sample. The pin rotates slowly backwards and forwards through an angle of 4.75°. Following generation of the first fibrin filaments between the pin and the wall of the test cup, the rotational range of the pin is reduced. The increasing restriction of the pin's movement is transferred to a graphical display, a plot that shows changes in the viscoelastic properties of the clot over time. The following parameters were recorded for the ROTEM tests: clotting time (CT (seconds); time from the start of the test until a clot firmness of 2 mm is detected), amplitude 10 (mm), the clot amplitude 10 minutes after the beginning of clotting) and the maximum clot firmness (MCF (mm)). We performed extrinsically activated thromboelastometric test (EXTEM), a test that uses rabbit brain thromboplastin as an activator, and fibrin-based thromboelastometric test (FibTEM), a test that assesses the fibrin-based clot using both extrinsic activation and addition of cytochalasin D to inhibit platelets' contribution to the formation of the clot (Figure 1). Reference ranges for the tests' parameters have been previously determined in a multi-centre investigation [27].

The standard protocol for ER management in our institution was followed. Blood for both ROTEM and routine laboratory testing was drawn immediately after placement of a central venous line on admission to the ER. Blood samples for ROTEM analysis were collected in a standard coagulation tube containing a 0.106 M citrate solution, resulting in a blood to citrate ratio of 9:1. ROTEM tests were performed according to the manufacturer's recommendations, and the analyses were started within five minutes of blood sampling. For prompt assessment of the patient's coagulation status, preliminary test results were obtained as early as five minutes after starting the analysis; the full results followed 10 to 20 minutes after starting the analysis. The ROTEM analyses were performed on admission to the ER and at the end of the operation (arrival at the ICU).

In parallel, laboratory analyses were performed as follows: fibrinogen concentration on the STA-R^® ^Analyzer (Stago Diagnostica, Asnieres, France); prothrombin time (PT) and activated partial thromboplastin time (aPTT) determined on Sysmex XE-2100 (Roche Diagnostics, Mannheim, Germany); haemoglobin, haematocrit and platelet count determined on Sysmex SF-3000 (Sysmex Corporation, Kobe, Japan); base excess determined on Roche OMNI^® ^S Blood Gas Analyzer (Roche Diagnostics, Mannheim, Germany). Standard laboratory analyses were performed on admission to the ER, on arrival at the ICU and 24 hours after admission to the ER.

At the beginning of our experience with ROTEM analysis, we observed that most of the major trauma patients showed a reduced MCF in the FibTEM test performed on admission to the ER. Low FibTEM MCF reflects reduced fibrinogen concentration or disturbed fibrin polymerization. To increase MCF, 2 to 4 g of fibrinogen concentrate (Haemocomplettan^® ^P, CSL Behring, Marburg, Germany) were administered as first-line therapy. A FibTEM MCF of 10 to 12 mm was chosen as the target value. Platelet concentrate was only transfused in patients not responding sufficiently to fibrinogen concentrate (i.e. absence of an adequate increase in MCF in the EXTEM test after the administration of fibrinogen concentrate).

Patients with recent intake of coumarins, as well as patients showing prolonged EXTEM CT (>1.5 times normal) received an additional 1000 to 1500 U PCC to augment thrombin generation. The following PCC products were used from 2005 to 2009: Beriplex (CSL Behring, Marburg, Germany), Octaplex (Octapharma, Vienna, Austria) and Prothromplex (Baxter, Vienna, Austria).

The target haemoglobin concentration during the operative procedure was 10 g/dL. In the postoperative phase, lower haemoglobin levels were tolerated.

Subjects' age and gender were noted, together with coagulation results, blood pressure, heart rate, temperature, Injury Severity Score (ISS), Revised Trauma Score and Glasgow Coma Scale (GCS) on admission. Predicted mortality for each patient was estimated using the TRISS methodology modified for intubated patients [28] and the RISC score [29]. Actual mortality until discharge from the hospital was also documented.

### Statistical analysis

For all parameters, normality of the data distribution was tested using the Kolmogorov-Smirnov test. Normally distributed results were expressed as mean ± standard deviation, and those distributed otherwise were expressed as median (25^th ^percentile, 75^th ^percentile). Depending on the underlying distribution, the Student's t-test or Mann-Whitney U Test was used to test for differences between survivors and non-survivors. Mortality rates (actual vs. TRISS or vs. RISC) were compared using the chi-squared test. The level of significance was set at *P *< 0.05.

## Results

From January 2005 until April 2009, 149 patients received five or more RBC units within the first 24 hours of ICU admission. Fifteen patients who died in the first hour after admission, most of whom arrived under cardio-pulmonary resuscitation, were excluded from the study, together with three patients who received no haemostatic therapy within the first 24 hours. Therefore, 131 patients were included in the analysis.

Patients' characteristics are listed in Table 1. There were 96 males and 35 females, with a mean age of 46 ± 18 years. The mean ISS was 38 ± 15. All but three patients received immediate emergency operative care. Statistically significant differences between survivors and non-survivors were observed: survivors were younger, had higher GCS scores, lower ISS and higher TRISS and RISC scores (i.e. higher predicted survival). The mean systolic blood pressure on admission to the ER was 88 ± 28 mmHg, with values of 100 mmHg or less in 106 patients. The mean base excess was -6.2 ± 3.5 mmol/l, with values of -10 mmol/l or less in 27 patients and -5 mmol/l or less in 79 patients. Thirty-three patients had operations for the control of abdominal, thoracic or vascular bleeding and 74 received immediate orthopaedic fracture fixation. Another 20 patients had combined orthopaedic and neurosurgical interventions. Three patients received no immediate emergency procedure.

**Table 1:** Demographic and clinical data

	**All patients**	**Survivors**	**Non-survivors**
	
N	131	99 (76%)	32 (24%)
Age (years)	46 ± 18	44 ± 17	52 ± 20*
Male (n [%])	96 (73%)	72 (73%)	24 (75%)
Weight (kg)	79 ± 14	79 ± 15	78 ± 11
BMI (kg/m^2^)	26 ± 6	26 ± 6	27 ± 6
GCS	11 ± 4	11 ± 4	8 ± 4*
ISS	38 ± 15	36 ± 15	44 ± 15*
RTS	6.2 ± 1.5	6.5 ± 1.3	5.1 ± 1.5*
TRISS	66 ± 31	74 ± 27	46 ± 31*
RISC	71 ± 27	79 ± 22	47 ± 29*

Data are presented as mean ± standard deviation, or as absolute and relative frequency. * *P *< 0.05, significant difference between survivors and non-survivors. BMI, body mass index; GCS, Glasgow Coma Scale; ISS, Injury Severity Score; n, number of patients; RISC, Revised Injury Severity Classification Score; RTS, Revised Trauma Score; TRISS, Trauma Injury Severity Score.

**Figure 2:**
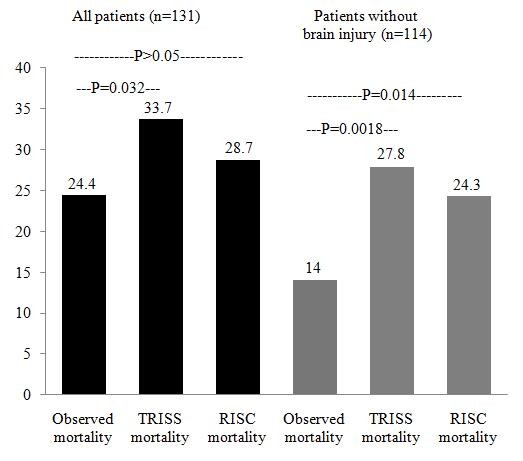
**Comparison of the observed mortality with the mortality predicted by the trauma injury severity score (TRISS) and by the revised injury severity classification (RISC) score**. A sub-analysis that excluded patients who died of untreatable brain oedema caused by severe brain injury was also performed.

The observed mortality was 24.4%, lower than the TRISS mortality of 33.7% (*P* = 0.032) and the RISC mortality of 28.7% (*P *> 0.05; Figure 2). After excluding 17 patients with traumatic brain injury, the difference in mortality was 14% observed versus 27.8% predicted by TRISS (*P *= 0.0018) and 24.3% predicted by RISC (*P *= 0.014).

**Table 2:** Thromboelastometric (ROTEM) parameters

	**Admission at ER**	**Admission at ICU**
	
EXTEM		
A10 (normal range 43 to 65 mm)	40 ± 10	35 ± 9
MCF (normal range 53 to 72 mm)	50 ± 8	46 ± 8
CT (normal range 35 to 80 seconds)	78 (63, 113)	71 (54, 105)
FibTEM		
A10 (normal range 7 to 23 mm)	5 (4, 7)	7 (5, 10)
MCF (normal range 9 to 25 mm)	6 (4, 8)	9 (6, 11)

Data are presented as mean ± standard deviation or as median (25th percentile, 75th percentile). A10, clot amplitude at 10 minutes after the beginning of clot formation; CT, clotting time; ER, emergency room; EXTEM, extrinsically activated thromboelastography test; FibTEM, fibrin-based thromboelastometric test; MCF, maximum clot firmness.

The ROTEM test results on admission to the ER and on arrival at the ICU are presented in Table 2. On admission to the ER, the mean MCF in EXTEM was 50 mm (normal range 53 to 72 mm). In the FibTEM test, the median MCF was 6 mm, lower than the normal range (9 to 25 mm). The median CT of EXTEM was within the normal range (78 seconds, normal range 35 to 80 seconds). On admission to the ICU, thromboelastometric parameters were comparable with the preoperative parameters.

**Figure 3:**
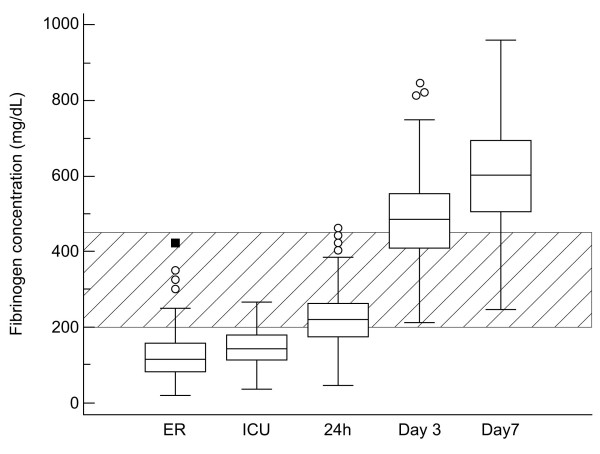
**Perioperative changes in plasma fibrinogen concentration**. Measurements were performed on admission to the emergency room (ER), on arrival at the intensive care unit (ICU), 24 hours after admission to the ER, on the third and the seventh postoperative days. The hatched area shows the normal physiological range of plasma fibrinogen concentration. The boxes represent the interquartile range, the lines represent the mean, and the whiskers extend to 95% confidence interval for the mean. The circles represent outside values, larger that the upper quartile plus 1.5 times the interquartile range, and the squares represent far out values, larger that the upper quartile plus three times the interquartile range.

The standard laboratory values are presented in Table 3. Mean plasma fibrinogen was 126 mg/dL on admission to the ER and 150 mg/dL on arrival at the ICU. The mean fibrinogen level only reached low-normal values 24 hours after admission to the ER (228 mg/dL, normal range 200 to 450 mg/dL; Figure 3).

**Table 3:** Standard laboratory parameters

	**Admission to the ER**	**Arrival at the ICU**	**24 hours after admission to the ER**
	
Haemoglobin (13.5 to 17 g/dL)	9.6 ± 2.8	9.6 ± 2.1	9.2 ± 1.5
Haematocrit (40 to 50%)	28 ± 8	28 ± 6	27 ± 4
Platelet count (150 to 350 *1000/μL)	166 ± 64	90 ± 49	79 ± 37
PT (11 to 13.5 seconds)	20.3 ± 8.3	22.6 ± 9.9	18.8 ± 3.2
aPTT (26 to 35 seconds)	53 ± 48	69 ± 47	49 ± 21
Fibrinogen (200 to 450 mg/dL)	126 ± 65	150 ± 50	228 ± 71

aPTT, activated partial thromboplastin time; ER, emergency room; PT, prothrombin time. Data are presented as mean ± standard deviation; normal range is indicated in parentheses.

In patients treated with fibrinogen concentrate, a median dose of 6 g was administered intraoperatively; the median cumulative dose during the first 24 hours was 7 g (Table 4). Patients who received haemostatic therapy in the ER due to the severity of bleeding received a median of 4 g as an initial dose. The maximum dose administered in the ER was 14 g. Further doses of 3 to 4 g were administered during the surgery and in the ICU. Only three of 131 patients did not receive fibrinogen concentrate. A median of six RBC units were transfused intraoperatively and a median of 10 RBC units were transfused during the first 24 hours. The median ratio of fibrinogen concentrate to RBC over the first 24 hours was 0.8 g per one unit. Despite the administration of high doses of fibrinogen concentrate, the mean postoperative fibrinogen plasma level was 150 mg/dL, which is below the normal range. In patients with prolonged CT in EXTEM, a median dose of 1800 U of PCC was administered during the operation and a median dose of 2400 U was administered during the first 24 hours (Table 4). A total of 30 patients received no PCC. Three patients with previous coumarin intake received only PCC for haemostatic therapy (between 2400 and 5400 U in 24 hours) and no fibrinogen concentrate.

**Table 4:** Haemostatic therapy and RBC transfusion

	Total administered until arrival at ICU		Total administered during 24 hours after admission to the ER	
		
	Number of patients treated	Dose	Number of patients treated	Dose
Fibrinogen concentrate (g)	123	6 (4, 9)	128	7 (5, 11)
PCC (U)	83	1800 (1650, 3100)	101	2400 (1800, 3600)
FFP (U)	6	10 (7, 10)	12	10 (9.75, 11.25)
PC (U)	22	2 (1, 2)	29	2 (2, 3)
RBC (U)	125	6 (4, 10)	131	10 (6, 13)

Data are presented as median (25th percentile, 75th percentile). Total number of patients = 131. ER, emergency room; FFP, fresh frozen plasma; PC, platelet concentrate; PCC, prothrombin complex concentrate; RBC, red blood cell concentrate.

The timing of the administration of coagulation factor concentrates is described in Table 5. Fifty-two percent of patients received these products within one hour of admission to the ER, and in most of these cases administration was within 30 minutes.

**Table 5:** Timing of the administration of coagulation factor concentrates

**Time of administration**	**Number of patients**
	
<1 hour after arrival in ER	68
1-2 hours after arrival in ER	34
2-6 hours after arrival in ER	24
6-24 hours after arrival in ER	5

ER, emergency room.

FFP was transfused in 12 patients, but always together with coagulation factor concentrates. Six of the 12 patients received FFP only postoperatively, in the ICU. Platelet concentrate was administered to 29 patients, 7 of whom received this treatment only in the ICU. Eight patients received recombinant activated factor VII and another seven received tranexamic acid/aprotinin.

## Discussion

In our retrospective analysis of 131 massively traumatised and bleeding patients, ROTEM-guided haemostatic therapy with fibrinogen concentrate as first-line haemostatic therapy and additional PCC was goal directed and fast. A favourable survival rate was observed.

The benefits of fibrinogen concentrate have been demonstrated in a variety of settings including trauma [19-21,30-33]. In massive bleeding, fibrinogen is the first factor that reaches critically low values [34,35]. Plotkin and colleagues showed in their study that reduced clot firmness was predictive for transfusion requirements [36]. Bolliger and colleagues investigated the minimum fibrinogen concentration above which clot formation normalises, and found that fibrinogen concentrations above 200 mg/dL are required [37]. In severe trauma, low fibrinogen levels are reached very early because of the dilutional effect of pre-hospital resuscitation. The mean preoperative fibrinogen plasma concentration in our patients was 126 mg/dL (shown by a FibTEM median MCF of 6 mm). Over the 24-hour period, a cumulative median dose of 7 g fibrinogen concentrate was applied. Despite this high dose, the median postoperative plasma fibrinogen level was 150 mg/dL, which is below the normal range of laboratory values.

A second argument that may support the safety of fibrinogen supplementation is that fibrin (known as antithrombin I, and formed from fibrinogen) acts by sequestering thrombin in the incipient clot, localising the further processes of clot formation [38,39]. Evidence of a remarkably low thrombogenic potential of fibrinogen concentrate has been recently presented by Dickneite and colleagues [40]. This study included experimental data from an animal model, and data from a 22-year pharmacovigilance program involving administration of more than 1,000,000 g of fibrinogen (Haemocomplettan P, CSL Behring, Marburg, Germany), equalling over 250,000 doses of 4 g. The reported incidence of thrombotic events possibly related to fibrinogen concentrate was 3.5 per 100,000 treatment episodes.

Fibrinogen concentrate therapy may also correct or compensate other haemostatic defects associated with haemodilution, such as decreases in platelet count or quality of fibrin polymerisation. *In vitro *and *in vivo *retrospective clinical investigations have shown that a high-normal fibrinogen level ensures satisfactory clot firmness at low platelet counts [41,42]. In the present study, although the median platelet count on arrival at the ER was within the normal range, subsequent haemodilution and consumption resulted in abnormally low values within the following 24 hours, despite administration of platelet concentrate. A strategy to reduce allogeneic blood product administration may be developed based on the possible compensatory effect of fibrinogen concentration in the presence of low platelet counts [41,42], but further clinical investigations are required to support this theory.

Volume replacement with crystalloids and colloids has a significant deleterious effect on clot properties, because fibrin polymerisation is impaired. This disturbance may be corrected by fibrinogen concentrate. For example, a study by Fenger-Eriksen and colleagues identified acquired fibrinogen deficiency as the main determinant of the dilutional coagulopathy induced by hydroxyethyl starches in patients undergoing total cystectomy [43]. As volume replacement in our trauma patients was also performed with such colloids, it is possible that fibrinogen concentrate therapy provided additional benefit by improving fibrin polymerisation.

PCC is recommended for emergency reversal of anticoagulation therapy [37], and its potential haemostatic value in bleeding patients without pre-existing coagulopathy has already been shown [22,23]. In our trauma patients, PCC was administered for the correction of the coagulopathy associated with the prolongation of the clotting time in the EXTEM test. PCC administration represented the second step of haemostatic therapy, and followed the administration of fibrinogen concentrate, which was aimed at correcting the decreased clot firmness. Combined administration of fibrinogen concentrate and PCC for correction of coagulopathy has already been investigated in a porcine trauma model [30], but until now this therapeutic approach has only been described in a single case report [44].

FFP is advocated for haemostatic therapy in patients with prolonged PT or aPTT [3]. However, there is a lack of evidence demonstrating that FFP controls blood loss [5,21]. Chowdhury and colleagues showed that 12 mL per kg bodyweight did not sufficiently increase the concentration of the coagulation factors [45]. Moreover, there are risks associated with FFP, such as transfusion-related acute lung injury, transfusion-related immunosuppression and pathogen transmission. Transfusion has been associated with increased morbidity and mortality [15-17,46-48] and these risks provide a rationale for minimising allogeneic transfusion. Furthermore, early and aggressive treatment of coagulopathy is essential in trauma patients, and it is questionable whether FFP treatment is compatible with this need. A study by Snyder and colleagues showed that the first FFP was given at a median of 93 minutes after arrival of the patient in the ER [49]. In contrast, 52% of the patients in our study received the first dose of fibrinogen concentrate within the first hour, most of them within 30 minutes. In addition, fibrinogen concentrate provides more effective increases in plasma fibrinogen concentrations within a short time interval. Within a few minutes 6 g of fibrinogen can be administered, and in our clinic coagulation factor concentrates are stored in the ER making them readily available. In contrast, the transfusion of FFP is time-consuming as delivery from the blood service and thawing are required, and there is a high volume load. Faster treatment with coagulation factor concentrates may be one reason for the favourable survival rate that we observed. It is also possible that the avoidance of the side effects of FFP contributed to this finding. Unlike FFP, coagulation factor concentrates undergo viral inactivation steps such as pasteurisation during their manufacture, minimising the risk of pathogen transmission.

It has been shown in the literature that 'blind' coagulation management (without point-of-care guidance) underestimates the real demand for coagulation factors in situations of severe bleeding [50]. Goal-directed coagulation treatment of severely bleeding patients necessitates quick and reliable coagulation monitoring, and a targeted therapeutic approach according to the test results [50]. Commonly used standard coagulation tests (PT and aPTT) are time-consuming and offer poor insight into the complex coagulopathy associated with high blood loss, factor consumption and haemodilution [51,52]. This makes them unsuited to guide therapeutic decisions in emergency settings. In contrast, ROTEM and thrombelastography support an accurate and timely assessment of not only the clotting initiation process, but also clot quality [24-26,53,54]. In animal models as well as in human studies, thrombelastography has been shown to be a reliable monitoring tool that detects clinically relevant clotting abnormalities associated with bleeding [55,56]. In a study that included 69 trauma patients, Kaufman and colleagues showed that only the ISS and the thrombelastography results were predictive of early transfusion requirements [25]. Furthermore, this methodology provides immediate results and consequently allows for the treatment to be tailored to patients' changing needs. The number of publications reporting or encouraging the use of ROTEM and thrombelastography for the guidance of haemostatic therapy is increasing continuously, including results in the trauma setting [31,44,57-61]. ROTEM tests have even been used to establish the dosage of haemostatic products [33,62] and to support the licensing procedure of fibrinogen concentrates [63]. Yet, although treatment algorithms based on ROTEM test results have been published, randomised controlled trials adopting such algorithms are not available at the moment [58,61].

Another advantage of the application of ROTEM- or thrombelastography-based algorithms is their potential to reduce transfusion and associated costs and their positive impact on patient outcome. The recently published 11th Health Technology Assessment reports on the clinical and cost-effectiveness of thrombelastography and thromboelastometry analysers compared with standard laboratory tests. This assessment recommends the use of these viscoelastic analyzers not only because of cost-effectiveness, but also because it can reduce the need for inappropriate transfusions and decrease blood product requirements. Overall, the report concludes that thrombelastography and thromboelastometry appear to have a positive impact on patient's health by reducing the number of deaths, complications and infections [64].

In the present study, observed mortality was significantly lower than the mortality predicted by TRISS (24.4% *vs *33.7%). The difference may be the result of multiple factors related to the management of trauma patients in our clinic, and therapy with coagulation factor concentrates could be one of these factors. The observation that the differences in mortality were even higher after exclusion of the patients with traumatic brain injury supports this assumption, because this group of patients was at risk because of uncontrollable brain oedema and not hypocoagulability. However, with the present study design, the impact of the haemostatic therapy algorithm on survival rate cannot be assessed separately. Furthermore, the clinical relevance of the lower observed mortality compared with the TRISS-predicted mortality must be weighed in view of the limitations of the TRISS score [65]. Although this score is used as a benchmark for mortality in the trauma setting, some trauma centres have reported observed mortality rates below those predicted by TRISS [65-67]. The greatest reduction in observed mortality versus the mortality predicted by TRISS (22% *vs *29%) was reported by Hirschmann and colleagues in a Swiss study of 172 university hospital patients with ISS of more than 15 [66]. The differences between the observed and predicted mortality may be influenced by inaccuracies of the TRISS score, the level of specialisation of the trauma care institution and by improvements in trauma care other than coagulation management. To circumvent these limitations, observed mortality was also compared with the mortality predicted by the recently developed RISC score that combines age, New Injury Severity Score, head injury, severe pelvic injury, GCS, PTT, base excess, cardiac arrest, and indirect signs of bleeding (shock, mass transfusion and low haemoglobin) [29]. In development and validation patient samples, the RISC score reached significantly higher values for receiver operating characteristic curves compared with TRISS [29]. When applied to the patients included in the present study, this score revealed mortality rates lower than TRISS-predicted mortality, in agreement with initial reports [29]. More importantly, RISC-predicted mortality was still higher than the observed mortality, and the difference reached statistical significance for the analysis that excluded patients with traumatic brain injury. Although this finding does not prove an association of the treatment algorithm with improved survival, it supports the assumption that trauma care incorporating ROTEM-based haemostatic therapy with coagulation factor concentrates is not detrimental to patients.

A number of factors may have contributed to the favourable survival rate: the promptness of the coagulation assessment (ROTEM results available in 10 to 15 minutes), the fast application of haemostatic therapy (half of the patients received goal-directed haemostatic therapy within less than one hour after arrival in the ER) and the high fibrinogen to RBC ratio (0.8 g: 1 unit over the first 24 hours). Regarding the latter factor, there have already been reports on the positive impact on survival that a high fibrinogen to RBC ratio may have. The retrospective analysis performed by Stinger and colleagues, which included 252 patients who received a massive transfusion (>10 units of RBCs in 24 hours), showed a lower incidence of death from haemorrhage in the group of patients receiving more than 0.2 g fibrinogen per RBC unit (mean amount of fibrinogen administered: 0.48 g fibrinogen per RBC unit) [19]. The amount of fibrinogen administered was calculated retrospectively as the total content of fibrinogen in the different types of blood products administered (i.e. FFP, platelet concentrates, cryoprecipitate, fresh whole blood and RBC). In our patients, the ratio fibrinogen to RBC was 0.8, nearly twice the ratio reported by Stinger and colleagues, but improvement of the fibrinogen level represented a central part of the therapeutic approach.

The main limitation of the present study is represented by its retrospective, uncontrolled nature that does not support an adequate estimation of the impact that therapy with coagulation factor concentrates may have had on mortality. A retrospective analysis of data before the introduction of thromboelastometry did not appear reasonable, because a variety of treatment protocols changed. The study did not assess outcome parameters apart from mortality, nor did it include comparison with non-ROTEM-guided haemostatic therapy. The present study was conducted over a fairly long time period (2005 to 2009), during which our experience with ROTEM-based coagulation therapy has increased and important changes in the clinic's standard transfusion protocols occurred. This is reflected by the fact that half of the 12 patients with FFP transfusion belong to the period 2005 to 2006. A clear reduction in intraoperative FFP transfusion occurred from 2006 and, in the following years, FFP has been mainly administered in isolated cases in the ICU at the intensivist's discretion. The same pattern was followed by therapy with recombinant activated factor VII: our records show no administration of this product in severe trauma patients after the middle of 2007.

## Conclusions

ROTEM-guided haemostatic therapy with fibrinogen concentrate as first-line haemostatic therapy and additional use of PCC was goal directed, efficacious, and quick to administer. Thromboelastometry allowed rapid and reliable diagnosis of the underlying coagulopathy. Given the favourable survival rate observed, the present data encourage prospective randomized studies based on this treatment strategy.

## Key messages

• The present study describes goal-directed haemostatic therapy of haemorrhage in severe trauma patients, in whom the administration of coagulation factor concentrates was tailored to correct the haemostatic defects identified by thromboelastometric analyses.

• The results show that coagulation factor concentrates (fibrinogen concentrate as first-line haemostatic therapy and additional PCC) can be used successfully in trauma patients with severe bleeding.

• Thromboelastometry (ROTEM) allowed rapid and reliable diagnosis of the underlying coagulopathy and guided the haemostatic therapy.

• Observed mortality appeared lower than the mortality predicted by the TRISS and by the RISC score.

• This treatment strategy may reduce allogeneic blood product transfusion, and prospective, randomized studies appear warranted.

## Abbreviations

APTT: activated partial thromboplastin time; CT: clotting time; ER: emergency room; FFP: fresh frozen plasma; GCS: Glasgow Coma Scale; ISS: Injury Severity Score; MCF: maximum clot firmness; PCC: prothrombin complex concentrate; PT: prothrombin time; RBC: red blood cell; SHOT: Serious Hazards of Transfusion; TRALI: transfusion-related acute lung injury; TRISS: trauma injury severity score.

## Competing interests

This study was performed without external funding. The article-processing charge is to be supported by the research group. Drs. Schöchl and Jambor have received honoraria as speakers from CSL Behring (manufacturer of fibrinogen concentrate and PCC) and Tem International GmbH (manufacturer of the ROTEM device). Dr. Solomon has received honoraria as a speaker and research support from Essex Pharma, CSL Behring, and Tem International GmbH. Dr. Kozek-Langenecker has received honoraria as a speaker and research support from Astra Zeneca, Baxter (manufacturer of PCC), B.Braun, Biotest, CSL Behring, Dynabyte, Ekomed, Fresenius Kabi, GlaxoSmithKline, Mitsubishi Pharma, NovoNordisk, and Tem International GmbH. All other authors declare that they have no competing interests.

## Authors' contributions

HS designed the study, wrote the manuscript, contributed to acquiring the data and revising the manuscript. UN contributed to the statistical analysis. GH, GS and WV contributed to the analysis of the data and to writing the manuscript. CJ contributed to acquiring the data. SKL contributed to designing the study and writing the manuscript. CS contributed to writing the manuscript, performed the statistical analysis and revised the manuscript.
